# Spatio-Temporal Structure of Hooded Gull Flocks

**DOI:** 10.1371/journal.pone.0081754

**Published:** 2013-12-10

**Authors:** Makoto Yomosa, Tsuyoshi Mizuguchi, Yoshinori Hayakawa

**Affiliations:** 1 Department of Mathematical Sciences, Osaka Prefecture University, Sakai, Osaka, Japan; 2 PRESTO, Japan Science and Technology Agency (JST), Chiyoda, Tokyo, Japan; 3 Center for Information Technology in Education, Tohoku University, Sendai, Miyagi, Japan; University of Arizona, United States of America

## Abstract

We analyzed the spatio-temporal structure of hooded gull flocks with a portable stereo camera system. The 3-dimensional positions of individuals were reconstructed from pairs of videos. The motions of each individual were analyzed, and both gliding and flapping motions were quantified based on the velocity time series. We analyzed the distributions of the nearest neighbor’s position in terms of coordinates based on each individual’s motion. The obtained results were consistent with the aerodynamic interaction between individuals. We characterized the leader-follower relationship between individuals by a delay time to mimic the direction of a motion. A relation between the delay time and a relative position was analyzed quantitatively, which suggested the basic properties of the formation flight that maintains order in the flock.

## Introduction

Some animals form groups and move collectively, such as schools of fish, flocks of birds, swarms of insects, and crowds of humans. Their motions vary according to species, the number of individuals, and their surroundings. Studies about such collective behaviors became popular after the pioneering work by Vicsek [1], forming a new class of research on *active matter* [2], [3].

Among them, flocks of birds exhibit interesting collective behaviors such as the V-shape formations of geese and the complex motions of starlings. Most previous studies are based on mathematical models, while works based on observational data are fewer, especially for birds that fly in flocks. Recently, it has become possible to obtain dynamic information from flying birds owing to the remarkable development of measuring devices such as global positioning systems (GPS), inertial sensors, and digital video cameras. Studies based on actual data thus tended to increase from the 2000s [4]–[9]. We need many more studies of actual data to understand individual’s dynamics and the interactions between individuals in flocks and to compare the results of mathematical models.

Current studies based on actual data are classified into 2 categories according to their observation methods. One is remote sensing [5], [6], [8] and the other is bio-logging [4], [7], [9]. For the former, a stereo camera system is a typical measurement method, which can capture the motion of flocks without touching the objectives. For the latter, small devices such as GPS and accelerometers are attached to individuals. They enable us to trace the dynamic behavior of each individual for a longer time than the former.

In this article, we used a portable stereo camera system and focused on hooded gull flocks that overwinter in Osaka, Japan. In comparison with birds of previous studies, the hooded gull’s body size is intermediate, and each individual’s motion in the flock exhibits varied behaviors. Hooded gulls flap slowly compared with the frame rate of a camera and their positions are fully separate from one another. Therefore, we can obtain each individual’s time series of position and velocity, including the effect the flapping motion. The shapes of some hooded gull flocks resemble V-shape formations like the skeins of geese, which are characterized by a distribution of the relative position of the nearest neighbor. The relation between the delay time to mimic a direction of a motion and a relative position was analyzed quantitatively.

## Materials and Methods

### Ethics Statement

Our study is based on videos of flying hooded gulls taken in the public space such as riverbanks and bridges each of which requires no specific permissions. Because the video recording procedure is passive and at least 20 m apart from the objectives, it does not interfere the activity of the birds. Our study does not involve endangered or protected species. We do neither touch nor feed the objectives, and our study follows the guideline for animal welfare in zoo and wildlife medicine of the Japanese Society of Zoo and Wildlife Medicine.

### Methods

Hooded gulls (*Larus ridibundus*) come to Osaka, Japan, to overwinter every year. They regularly come and go between Osaka Bay and town areas along Yamatogawa River at sunrise and sunset. Therefore, it is convenient to observe their flight performance.

To reconstruct the 3-dimensional position of each individual, we used a portable stereo camera system [8]. Two commercial cameras (Canon, EOS 5D MarkII and Canon, FE 50 mm F1.4 USM) were positioned on a rigid bar 1-m apart and mounted on a portable tripod. These cameras take 30 pictures per second. We used a laser ranging system (Nikon, LASER 550AS) to measure an elevation angle between an optical axis of a photographing lens and the horizontal plane and the distances of reference points. We recorded several videos from riverbanks and bridges. Using a disparity between the images taken by the two cameras and the elevation angle, we reconstructed 3-dimensional position data of each individual. We used a phase only correlation method to get a sub-pixel accuracy of the disparity between the images [10]. Consequently, the distance resolution was higher than 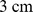 for objectives 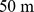 apart from the cameras, which is checked by comparison with the distances measured by a rule and by our system as shown in Fig. S1. The typical distance between the stereo camera and the birds ranged from 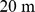 to 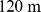. The typical time in which we reconstructed the 3-dimensional data for each individual in one video was several seconds because of an angle view limitation. In this paper we analyzed 7 flocks labeled from A to G, whose information is summarized in Table 1. A typical video of a flock motion and the reconstructed trajectories are attached as Video S1 and Data S1. Note that each individual came in and out the angle view of the cameras at different timing. Thus, the number of individual in one snapshot may not be the same as the total individual number 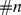. We show sample of snapshots and a reconstructed trajectory of each individual in Fig. S2.

**Table 1:** Analyzed flocks information.

Flock label	A	B	C	D	E	F	G
Individual number #*n*	35	23	22	15	5	3	3
Observation duration [s]	9.77	6.77	5.63	4.80	5.37	7.73	4.93
Pearson’s r	−0.938	−0.461	−0.899	−0.946	−0.955	−0.999	−0.999
Pearson’s N	54	432	342	38	12	6	6

Note that the observation duration is a video time length. Because of an angle view limitation, time length of some individuals is shorter than the observation duration. Pearson’s r and N are the correlation coefficient of 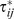 vs. 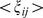 in Fig. 6(a) and the analyzed pair number of each flock, respectively. Pearson’s P of each flock is less than 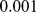. Note that possible number of independent data is 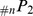 because 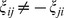. N is, however, smaller than it in some groups because there are some pairs which do not appear in the same frame.whose time series are not enough to calculate 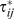.

The number of individuals in one snapshot of the analyzed flocks was 23 at most, and the matching of left and right images was performed manually. The typical distance between the nearest neighbor pairs is approximately 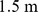, which is several times larger than the distance one individual moves in one frame (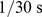). Therefore, the motion of each individual can be traced. If we could not reconstruct an individual’s position data from a pair of snapshots by overlapping other individuals’ images, we interpolated the data using the before and after snapshots.

## Results

### Single Individual Analysis

**Figure 1:**
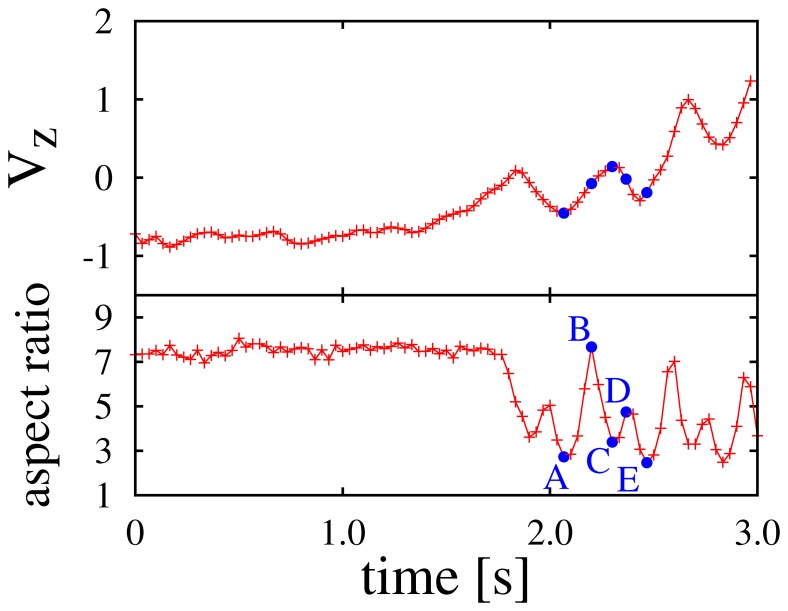
Time series of a vertical component of velocity *V_z_* (top) and aspect ratio (bottom). The flight mode switches from gliding to flapping at around *t* = 1.7 s, where both quantities begin to oscillate. Indexes correspond with those in Fig. 2.

**Figure 2:**
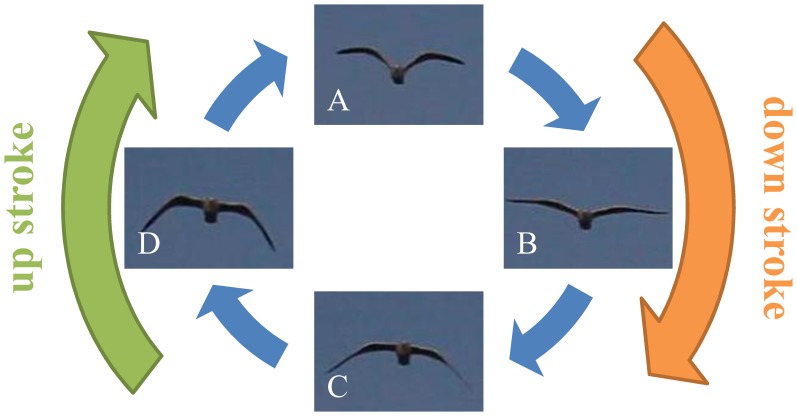
Individual images of the flapping motion.

Table 2 presents the basic data of *L. ridibundus*. Due to the large body length and a slow flapping frequency, oscillating data due to the flapping motion was extracted from the position time series. Thus, the velocity time series also included the effect of the flapping motion. Figure 1 (top) illustrates the time series of the vertical component of the velocity. The data in Fig. 1 were obtained by an isolated flight of a single individual 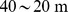 away from the cameras, and exhibited the switching of flight modes from gliding to flapping (Video S2, Data S2 in Supporting Information). This switching between 2 modes was also characterized by the shape variation of the individual’s image. In order to characterize the shape variation according to the flapping motion quantitatively, we measured an aspect ratio obtained from an ellipse that fit the individual’s image using *ImageJ*. In Fig. 1 (bottom), the individual begins to flap after approximately 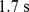. The correspondence between the oscillation of the velocity component and the shape variation by the flapping motion is confirmed by these 2 graphs. Figure 2 depicts individual images of the flapping motion. The indexes in the images correspond to Fig. 1. Image A is a snapshot in which the wing tips are at the top and image C is a snapshot in which the wing tips are at the bottom. Note that the shape of bird at E in Fig. 1 is almost the same of A. Thus, the motion from A to C through B is a downstroke, and the motion from C to E through D is an upstroke. Corresponding with this motion and Fig. 1, we confirmed that the vertical component of the velocity increases between the downstroke. Furthermore, the maximum of the aspect ratio during the downstroke was larger than that of the upstroke. It suggests that individuals stretch out their wings during the downstroke and fold them during the upstroke to increase the lift force during the wingbeat cycle [11].

**Table 2:** Basic physical values [11] and observation data.

Quantity	Value	Observation data
Mass	0.285 kg	
Wing span	0.967 m	
Wing area	0.0992 m^2^	
Velocity	9.50 m/s, 15.7 m/s†	10.0∼15.0 m/s
Wingbeat frequency	4.04 Hz	3.33∼4.29 Hz

† 9.50 m/s is minimum power speed and 15.7 m/s is maximum range speed. Note that the velocity of basic physical values are true air speed and the observational velocity is ground speed, which is affected by airflow.

Hereafter, we focused on the trend position and velocity obtained by averaging the oscillating component by flapping. All analyzed data in this article except Fig. 1 were averaged for each wingbeat period whose typical value was 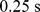.

### Relation between the Nearest Neighbor Individual

**Figure 3:**
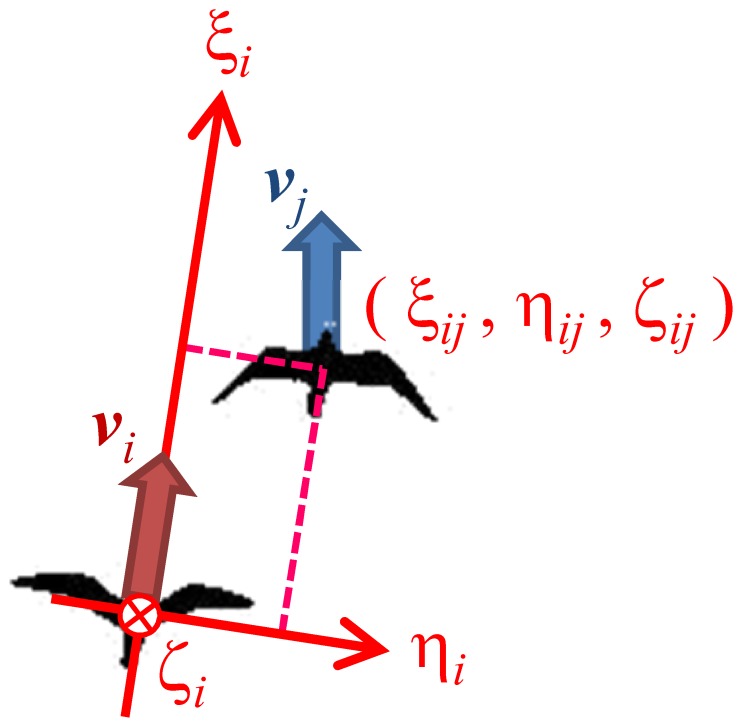
Illustration of the individual coordinates. The 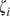-axis is orthogonal to these 2 axes. 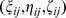 indicates the relative position from individual *i* to individual *j*.

In this system, we obtained the time series of each individual’s dynamic quantities in camera-based coordinates. We, however, introduced “individual’s coordinates” of the *i*-th individual based on the direction of the *i*-th individual’s motion (Fig. 3) to analyze the data from the viewpoint of interacting self-propelled particles. Let 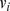 be the trend velocity of the *i*-th individual. The 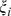-axis is taken to be the trend direction of movement of the *i*-th individual. The 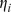-axis is parallel to the horizontal plane and orthogonal to the 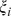-axis. The 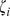-axis is orthogonal to the 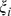
- and 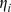-axes. The relative position of the *j*-th individual from the *i*-th individual is represented by 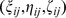. Two things should be notes: (i) 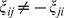 because the trend moving directions of the *i*-th and *j*-th individuals do not coincide generally. (ii) The coordinates are based on the individual’s motion, which should be distinguished from the direction of its body, such as the rostral, dorsal, and lateral direction.

**Figure 4:**
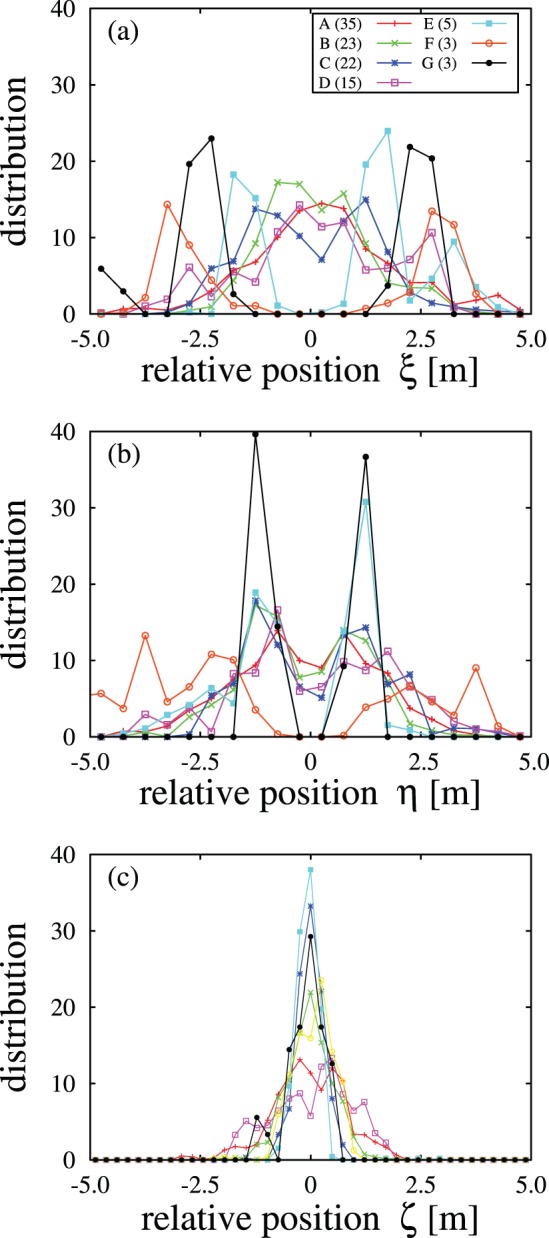
Distributions of relative position of the nearest neighbor in flocks. The numbers after indexes A∼G denote the number of individuals in the flock.

Using the individual’s coordinates, we analyzed the relative position of the nearest individual in flocks. Figure 4 depicts the distribution functions of the relative position of the nearest neighbor to the individual’s coordinates for 7 flocks. The width of distribution function of the 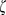 direction is narrower than others. Therefore, the flock is thin; roughly speaking, it has a 2 dimensional structure. A double-peak structure with a width spanning approximately the wingspan length (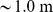) of the 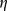 direction is robustly observed regardless of the distributions of the other directions. It suggests that individuals avoid the position just behind another individual due to an aerodynamic interaction between them. Furthermore, there is maximum aerodynamic advantage at the wingspan length side behind an individual [8], [12]. The distribution of the 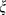 direction differs by flock and they are roughly classified into 2 types, i.e., single or double peak structure. The fact that both distributions of the 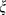 and 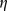 directions have double peak structures suggests that the configuration of the flock resembles the V-shape formations of skeins of geese. The single peak structure of the distribution of the 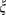 direction and the double peaks for the 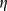 direction denote that individuals fly side by side, thus the configuration of the flock is not V-shaped. In the final section, we will return this point.

### Leader-follower Relationship

Next, we characterized the leader-follower relationship between individuals with the following 4 steps. (i) Measure trend velocity 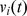 of the *i*-th individual. (ii) Calculate correlation function 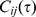 of the direction of movement between all pairs of *i*-th and *j*-th individuals.
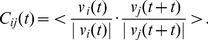
(1)

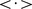
 denotes the average for time 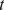. (iii) Define delay time 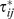 for all pairs that 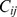 derives the maximal value at 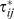. (iv) 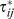 determines the leader-follower relationship between the *i*-th and *j*-th individuals, i.e., if 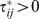, the *i*-th individual is being followed by the *j*-th individual. Note that 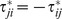.

**Figure 5:**
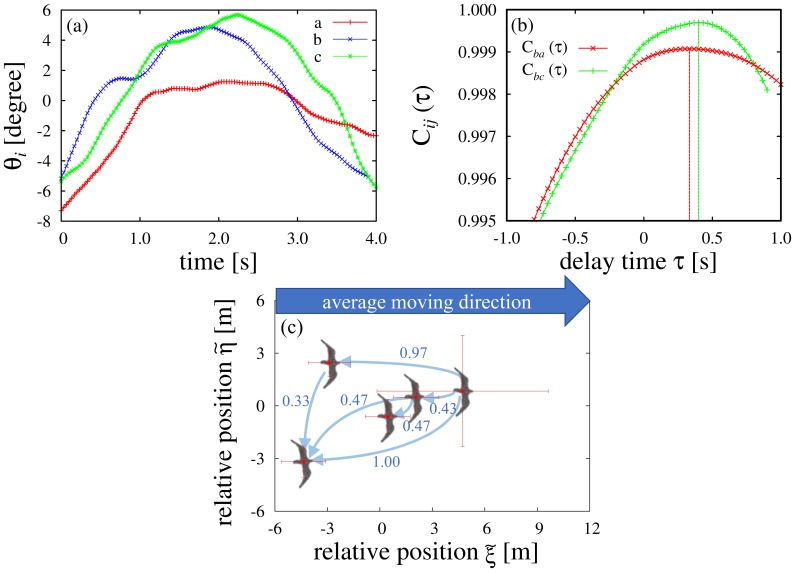
A typical example of a delay time in Flock E. (a) Time series of the angle between direction of movement of individuals and the average direction of movement of the flock. (b) Two correlation functions 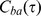 and 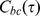. (c) The leader-follower relation is drawn on the average position of the flock. The arrow is drawn from the leader to the follower. The numbers attached to each arrow is the delay time. Only arrows for pairs with 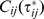 which exceeds a threshold value (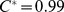) are drawn. The vertical and horizontal error bars of each individual show the standard deviations in the observation time.

Figure 5(a) is the time series of the angle between the direction of movement of individuals and the average direction of movement of the flock. Individual *b* changes the direction of movement earlier than individuals *a* and *c*. Figure 5(b) illustrates 2 correlation functions: 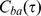 and 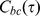. In this case, 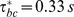 and 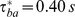. Therefore, both individuals *a* and *c* follow individual *b*. The similarity of a behavior is represented by the value of 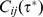. The fact that 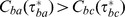 is interpreted in that *a* mimics *b* better than *c* does. We constructed the leader-follower network from the set of delay times between pairs. In Fig. 5(c), the leader-follower network is superimposed on the average relative position of all individuals in the flock. The origin (0,0) is the center of the flock and the 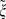-axis is the average direction of the flock motion. the 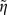-axis is orthogonal to the 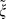-axis and parallel to the horizontal plane. The thin arrows illustrate the leader-follower relationship between individuals. Namely, if 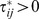, the arrow is drawn from individual *i* to individual *j*. All arrows head from the front to the back of the flock. Thus, Fig. 5(c) suggests that rearward individuals tend to follow forward individuals.

**Figure 6:**
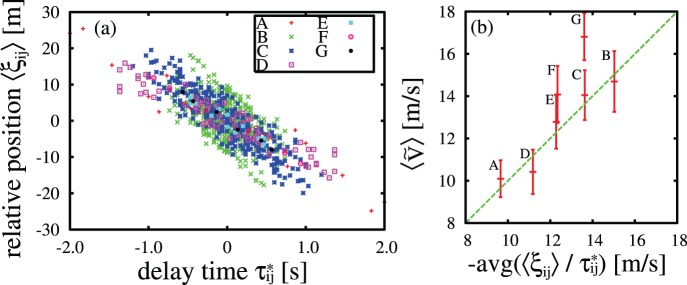
The relation between the delay time, the relative position and the averaged velocity. (a) The relation between the delay time and the relative position of each flock. (b) The relation between slopes of (a) and the averaged velocity both for individuals and observation time for each flock. Error bars denote the standard deviation during the observation time. Dashed line is the diagonal line as a visual guide.



**Figure 7:**
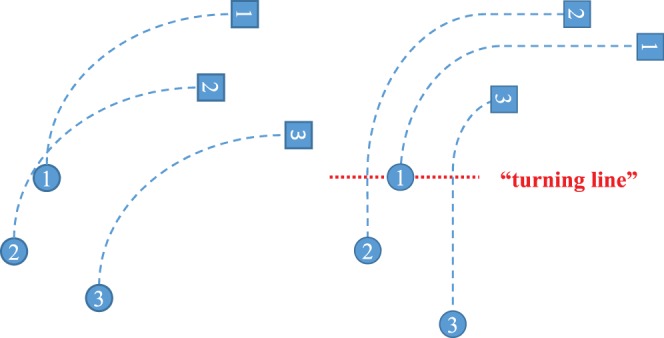
Illustration of turning flight. (a) Simultaneous turning and (b) turning with delay. Numbered circles and squares represent individuals’ positions before and after turning, respectively. Dashed lines are trajectories of each individuals. Red dotted line indicates “turning line” perpendicular to the direction of movement and past at which the preceding individual started to turn.

Figure 6(a) depicts the relation between the delay time 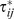 and 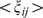, a temporal average 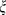-directional relative position of individual *j* to individual *i*. 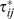 and 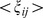 have a negative correlation and their slopes are distributed. For each flock, we estimated the slopes, 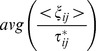, using the principal component analysis and compared them with the averaged velocity both for individuals and observation time, 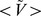. 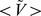 ranges 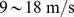 because it depends on surrounding conditions such as wind, height, and situation. We show the correlation coefficient in Table 1. Figure 6(b) illustrates the relation between 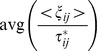 and 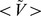 of each flock. It suggests the relationship

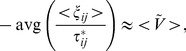
(2)except in flocks consisting of small members (F and G). It implies that followers do not turn simultaneously with the preceding individuals but at a “turning line” perpendicular to the average direction of movement in which the preceding individuals turn, as shown in Fig. 7.

## Discussion

Our system detected the flapping motion of individuals from the oscillating component of the velocity, 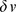, which contains much more information than the aspect ratio does. For example, 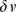 possesses 3-dimensional components, and is measurable where the aspect ratio is difficult to measure. In Fig. 1, there is a weak oscillating component of 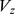 in the gliding mode. However, the amplitude of 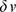 is less than flapping flight, which is distinguishable.

The distribution of the 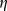 direction of the relative position of the nearest neighbor demonstrates two features: the avoidance region just behind the preceding individuals and the preference positioning separated by a wingspan. The former is observed in other species of birds such as geese [8] and starlings [5]. Thus, it is expected to be a common feature for many kinds of birds. The latter suggests their strategy of using upward flow created by a preceding individual. In this case, they prefer to locate the diagonally behind the preceding individual. If this positioning is succeeded by the next followers one after another, so-called V-shape flock is expected to be formed, which is well observed for large size of birds like geese or cranes. For smaller birds such as starlings, however, such a regular behavior is hardly observed. It is an open problem what makes this difference. The aerodynamic effect may be relatively weaker than another attracting effect [9] or internal degrees of freedom such as heading vector may play a crucial role [13].

The difference of the distribution of the 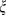 direction may relate to the difference of the flock shape. When we recorded the videos, we obtained various flock shapes in our visual impression, such as “loose” and “ordered” flocks. The single peak structure of the distribution of the 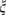 direction represents the breakdown of the V-shape formation. The thickness of the flocks variance of the distribution of the 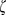 direction has a weak correlation with the type of distribution of the 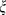 direction. The relation suggests that when individuals overtake others, they move out of the flock “plane”. This may cause the difference of the flock shapes. This problem also remains unresolved.

In terms of the distributions of the nearest neighbors, for example, flock A is not ordered compared with flock E. Both flocks A and E, however, satisfy Eq. 2. Thus, Eq. 2 may be the minimum condition for hooded gulls to fly collectively. In Fig. 6(b), flocks F and G deviate from the diagonal line. We think that this deviation comes from the fluctuation due to the smallness of the flock member.

To maintain the formation flight, in which individuals keep their order in flocks, individuals should adjust their relative position by using an appropriate delay time. Figure 7 illustrates a simultaneous turning (left) and turning with delay (right). If the followers turn simultaneously, the configuration of the flock should change by turning. In some cases, such as starlings or rock doves flying around their roost, the relative position in the flock changes [6], [14], which is different from the formation flight observed in geese. Therefore, the delay is an important necessary condition to keep flight formation.

From the viewpoint of *active matter*, these behavior can be compared with other kind of animals or species as a collective behavior of self propelled particles. For individuals moving in a fluid, the influence of the wake produced by the preceding individuals is important and there are some preference or avoidance positions like Fig. 4(b), e.g., a slipstreaming of automobiles and bicycles or a diamond formation of fish schooling [15]. These behaviors are due to the vortex structure of the preceding individuals’ wake. And energetic analysis with considering their differences such as Reynolds number, Strouhal number and the driving mechanism should be one of the key ways [16]. And, the relation expressed by Eq. 2 is related to maintain an internal structure of the flock, a formation flight. Therefore, it may be observed for the group behavior of keeping the relative position. Anyway, further studies with more detail data for various kinds of birds or other animals are welcomed.

## Supporting Information

Figure S1
**Distance resolution of the system.** We set up an objective 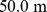 apart from the camera system. We use this objective for the reference point, i.e., we adjust the parameter of the system so that the reconstructed distance of the objective to be 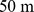. Then, the distance between the objective and the camera system is changed to 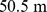 step by step with an interval 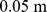, and the distance measurement is performed by our system for each step. The horizontal axis is the relative distance measured by a rule from the first objective 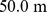 apart and the vertical axis is that measured by our system. All the error fall within the range of 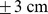.(PDF)

Figure S2 **A typical example of images and reconstructed data (flock C, #** ***n*** **= 22).** (a) Left and right image taken at the same timing. This pair can be a stereogram with the parallel view method. (b) Reconstructed flock under the same perspective in (a). (c) Reconstructed trajectories of each individual. X,Y and Z form the coordinates system based on the camera pairs. The duration of the trajectories is 3 seconds. Each point corresponds to the timing of (a). The same colors are used in (b) and (c).
(PDF)

Video S1 **A typical video of a flock motion (flock C).**
(MOV)

Video S2 **A video of a single flight which include both gliding and flapping motion (** **Figure 1** **and** **2** **).**
(MOV)

Data S1 **Trajectory data of each individual in flock C (Video S1).**
(TXT)

Data S2 **Time series of a trajectory and an aspect ratio (Video S2).**
(TXT)
