# Supplementary material for: Spatio-Temporal Structure of Hooded Gull Flocks
Source: PLoS One. 2013 Dec 10;8(12):e81754. doi: 10.1371/journal.pone.0081754 (PMC3858251; doi:10.1371/journal.pone.0081754)

Figure S1. Distance resolution of the system. We set up an objective 50.0 m apart from the camera system. We use this objective for the reference point, i.e., we adjust the parameter of the system so that the reconstructed distance of the objective to be 50.0 m. Then, the distance between the objective and the camera system is changed to 50.5 m step by step with an interval 0.05 m, and the distance measurement is performed by our system for each step. The horizontal axis is the relative distance measured by a rule from the first objective 50.0 m apart and the vertical axis is that measured by our system. All the error fall within the range of ± 3 cm.

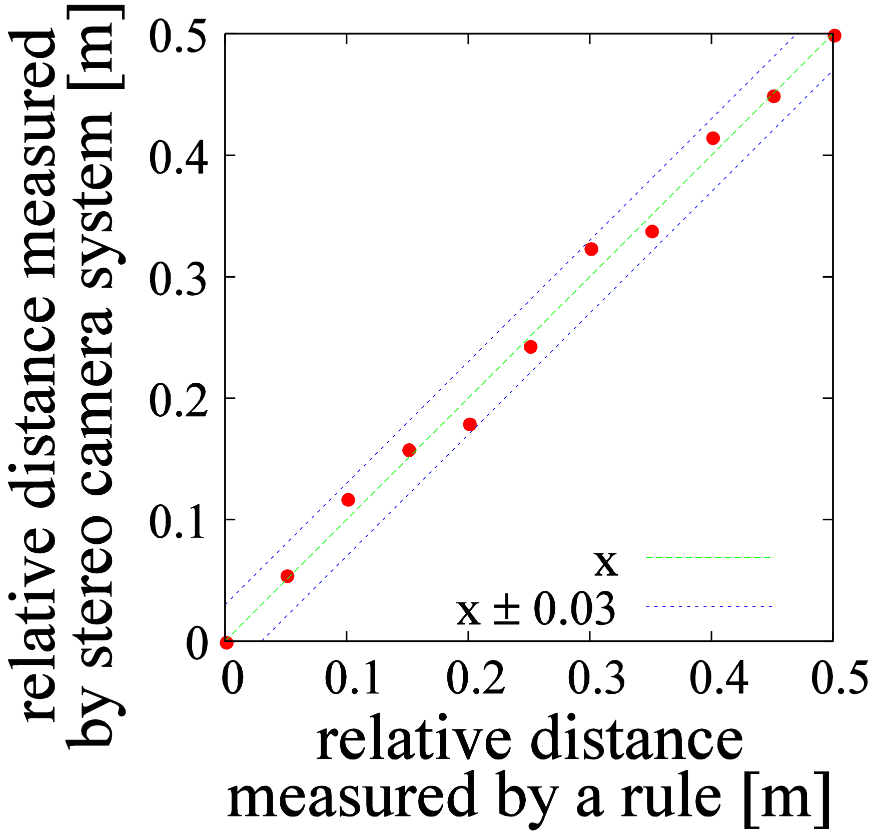

Supplement: Figure S1 — Distance resolution of the system. We set up an objective apart from the camera system. We use this objective for the reference point, i.e., we adjust the parameter of the system so that the reconstructed distance of the objective to be . Then, the distance between the objective and the camera system is changed to step by step with an interval , and the distance measurement is performed by our system for each step. The horizontal axis is the relative distance measured by a rule from the first objective apart and the vertical axis is that measured by our system. All the error fall within the range of . (PDF) [file pone.0081754.s001.pdf]
