# Supplementary material for: Spatio-Temporal Structure of Hooded Gull Flocks
Source: PLoS One. 2013 Dec 10;8(12):e81754. doi: 10.1371/journal.pone.0081754 (PMC3858251; doi:10.1371/journal.pone.0081754)

Figure S2. A typical example of images and reconstructed data (flock C, #n = 22).

(a) Left and right image taken at the same timing. This pair can be a stereogram with the parallel view method.

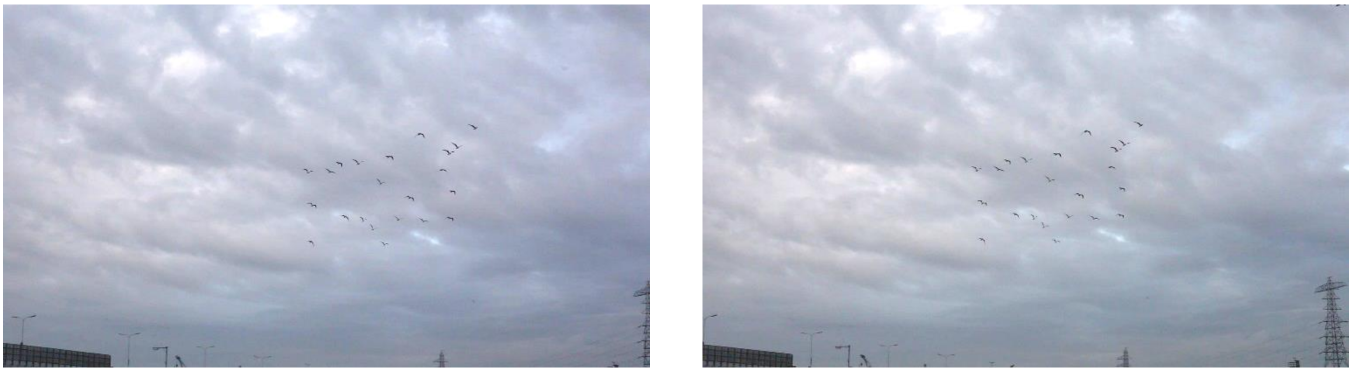

(b) Reconstructed flock under the same perspective in (a).

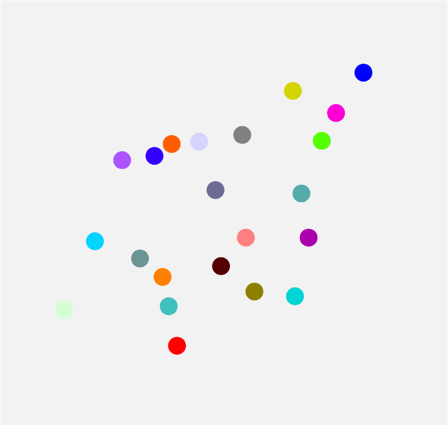

(c) Reconstructed trajectories of each individual. X,Y and Z form the coordinates system based on the camera pairs. The duration of the trajectories is 3 seconds. Each point corresponds to the timing of (a). The same colors are used in (b) and (c).

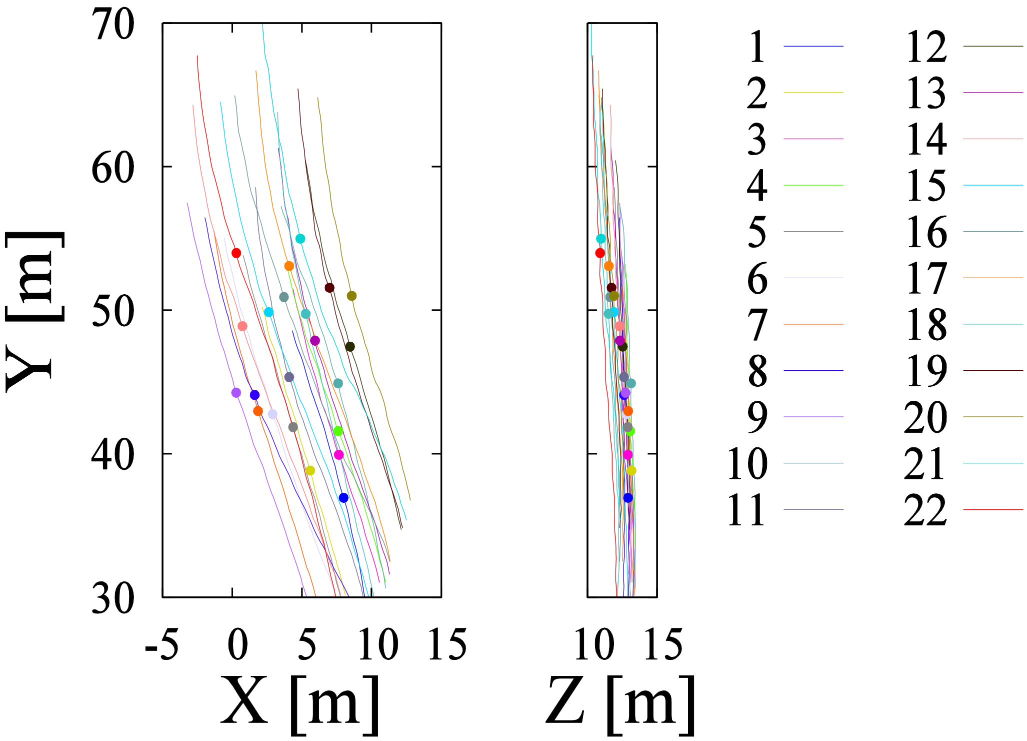

Supplement: Figure S2 — A typical example of images and reconstructed data (flock C, # n = 22). (a) Left and right image taken at the same timing. This pair can be a stereogram with the parallel view method. (b) Reconstructed flock under the same perspective in (a). (c) Reconstructed trajectories of each individual. X,Y and Z form the coordinates system based on the camera pairs. The duration of the trajectories is 3 seconds. Each point corresponds to the timing of (a). The same colors are used in (b) and (c). (PDF) [file pone.0081754.s002.pdf]
